# Association of AdipoQ gene variation (rs1501299) and oxidative stress with cardiovascular disease in North West Indian population of Punjabi women

**DOI:** 10.5937/jomb0-24704

**Published:** 2021-01-26

**Authors:** Jyot Amrita, Mridula Mahajan, A.J.S. Bhanwer, Kawaljit Matharoo

**Affiliations:** 1 Sri Guru Ram Das Institute of Medical Sciences and Research, Department of Biochemistry, Amritsar, Punjab, India; 2 Government Medical College, Department of Biochemistry, Amritsar, Punjab, India; 3 Guru Nanak Dev University, Department of Human Genetics, Amritsar, Punjab, India

**Keywords:** AdipoQ gene, cardiovascular disease, MDA, SOD, SOD, MDA, kardiovaskularno oboljenje, AdipoQ gen

## Abstract

**Background:**

Till to date whether adiponectin AdipoQ gene variation (rs 1501299) is associated with cardiovascular disease, still remains controversial. Therefore, we aimed to relate the SNP (rs1501299) of adiponectin gene and oxidative stress in context to CVD in Punjabi women of North West India.

**Methods:**

In the present case-control study menopausal women with CVD as cases (n=265) and menopausal women without CVD as controls (n=258) were recruited. Genotyping of rs1501299 single nucleotide polymorphism of adiponectin gene was carried out by RFLP-PCR analysis. Biochemical parameters were analyzed according to the standard procedures.

**Results:**

Distribution of homozygous TT genotype of normolipidemic (p=0.001) and hyperlipidemic (p=0.001) women with CVD was significantly more frequent as compared to women without CVD. rs1501299 T allele carriers with CVD also showed significant (p=0.001) higher frequency distribution as compared to women without CVD. Under recessive model of inheritance TT mutant type homozygotes conferred ~9 fold higher risk [p=0.001; OR= 9.60 (2.92-31.58)] towards CVD susceptibility for MDA>1.50; ~11 fold higher risk [p=0.007; OR= 11.11 (1.49-82.83)] towards CVD for LDL carbonyl protein>15.04 and ~9 fold higher risk [p=0.001; OR= 9.75 (2.30-41.22)] towards CVD susceptibility for SOD≤5.55. Under logistic regression analysis oxidative stress and TT genotype were significantly correlated with CVD.

**Conclusions:**

Our study revealed significant association of AdipoQ (rs1501299) gene polymorphism and oxidative stress with cardiovascular disease in Punjabi women of North West India. However, additional studies are required to support these findings.

## Abstract (sr)

**Uvod:**

Do današnjih dana još uvek je nejasno da li su promene adiponektin AdipoQ gena (rs 1501299) udružene sa kardiovaskularnim oboljenjem. Iz toga razloga ispitivan je odnos SNP (rs1501299) adiponektin gena i oksidativnog stresa u vezi sa kardiovaskulanim oboljenjem (CVD) u žena iz Pendžaba u Severozapadnoj Indiji.

**Metode:**

U ovom kontrolisanom ispitivanju obuhvaćene su žene u menopauzi sa CVD (n = 265) i žene u menopauzi bez CVD kao kontrole (n = 258). Genotipizacija rs1501299 nukleotidnog polimorfizma adiponektin gena izvođena je primenom RFLP-PCR analize. Biohemijskim parametri određivani su pomoću standardnih procedura.

**Rezultati:**

Raspodela homozigota TT genotipa normolipidemijskih (p = 0,001) i hiperlipidemijskih (p = 0,001) žena sa CVD bila je mnogo značajnija u poređenju sa ženama bez CVD. rs1501299 nosioci alela sa CVD pokazali su takođe značajno (p = 0,001) višu raspodelu frekvencije u poređenju sa ženama bez CVD. Recesivni model nasleđivanja TT mutiranog tipa homozigota pokazao je 9 puta viši rizik [p = 0,001; or = 9.69 (2,92-31,58)] prema CVD osetljivosti za MDA>1,50; ~11 puta viši rizik [p = 0,007; or = 11,11 (1,49-82,83)] prema CVD za LDL karbonil protein > 15,04 i ~9 puta veći rizik [p = 0,001; or = 9,75 (2,30-41,22)] prema CVD osetljivosti za SOD 5,55. Ispod logističke regresione analize oksidativni stres i TT genotip bili su u značajnoj korelaciji sa CVD.

**Zaključak:**

Ispitivanje je pokazalo značajnu vezu genskog polimorfizma AdipoQ (rs1501299) i oksidativnog stresa sa kardiovaskularnim oboljenjem u Severnozapadnih indijskih Pendžabskih žena. Međutim, neophodna su i dodatna ispitivanja kako bi se ove činjenice potvrdile.

## Introduction

Cardiovascular disease (CVD) constitutes a major health problem in many parts of the world and is an important cause of morbidity and mortality. CVD is a multifactorial disease as both genetic and environmental factors contribute to its aetiology. In 2013, the leading cause of death in women 65 years of age was diseases of heart. Cardiovascular disease was the cause of death in 398086 females. Age -adjusted death rates for females were 1 in 3.2 females for CVD [1]. INTERHEART and INTERSTROKE studies have reported that the associated risk factors with CVD are as important in India as in other populations of the world [2]. In today's society where women play multiple roles as caregivers, homemakers and breadwinners, unhealthy life style practices, lack of exercise, consumption of fat rich diet and chronic low level stress may contribute to risk factors leading to CVD. Moreover, cardiovascular disease becomes more apparent in women at menopause. However, in addition to modifiable risk factors, genetic factors can also predispose individuals to CVD.

Adiponectin is a polypeptide hormone that is produced and secreted into the blood by mature adipocytes. Adiponectin influences a number of metabolic processes particularly glucose and fatty acid metabolism in the liver and muscles. It plays an important role in anti-inflammatory, antiatherosclerotic and insulin-sensitizing activities [3]. Human adiponectin gene referred as *AdipoQ* previously called *Acrp 30* (adipocyte complement-related protein of 30kDa), *APM1* (adipose most abundant gene transcript) and *GBP28* (gelatin binding protein of 28kDa) is located in chromosome 3q27, has a structural homology with collagen VII and IX, complement factor CIq and TNF family [4]. The most extensively studied polymorphism within the adiponectin gene is *ADIPOQ* +276G>T (rs1501299) variant located in intron 2, and is a result of a G to T substitution [5]. The variants of *AdipoQ* gene were found to be associated with obesity, metabolic syndrome markers and cardiovascular disease [6] [7].

Only a few previous studies have explored the association of *AdipoQ* (rs1501299) polymorphism and the concomitant presence of cardiovascular disease with nearly no reports from Northern Punjabi population of India particularly in women at menopausal age. Studies have also showed association of *AdipoQ* polymorphism with Type 2 Diabetes [8] [9]. Moreover, the overall reported associations of these polymorphism and cardio metabolic disease have been diverse. The small numbers and varying populations may account for the controversial results. Thus, the present study was proposed with a hypothesis that rs1501299 variation in *AdipoQ* gene is among the genetic factor predisposing the female menopausal population of Punjab to CVD. Menopausal women were the choice for the subjects in the present study because at this stage many hormonal variations such as, decline in the estrogen levels occur, which may cause imbalance in the oxidative processes and also may increase the risk of metabolic diseases. We also aimed to relate the soundly studied SNP (rs1501299) adiponectin gene polymorphism with oxidative stress in context to CVD in Punjabi women of North West India.

## Materials and Methods

### Study population

In the present case-control study 265 menopausal women with CVD as cases (mean age 44±4 years) and 258 menopausal women without CVD as controls (mean age 45±4 years) were recruited. Cases from in-patient and out-patient Department of Medicine of Sri Guru Ram Das Institute of Medical Sciences and Research, Amritsar, Punjab (India) were enrolled. The diagnosis of cardiovascular disease of the patient was made by the physician on the basis of history, clinical symptoms, supportive by documented ECG findings and angiography (where ever required for the diagnosis). Menopausal women from general population having no evidence of CVD or any past history of the disease were considered as controls.

For the entire participants face-to-face interview using a standard questionnaire was followed which included written informed consent of all the participants taken before sampling and detailed clinical history including general, physical and systemic examination. The study was approved by the institutional ethics committee.

*Inclusion criteria*: All menopausal women were included (both natural and surgically induced menopause). Initially, a pilot study of 50 subjects was done to compare the levels of prooxidants, antioxidants, and lipid profile between natural menopausal women and surgically induced menopausal women. No difference in the levels was observed. Therefore, we combined both the groups as a whole emphasizing the comparison only between menopausal women with CVD and menopausal women without CVD. Both the groups were matched for the age at menopause.

*Exclusion criteria*: Women suffering from any chronic disease, acute infections and renal disease were excluded. Women on hormonal therapy, any antioxidant supplements and lipid lowering drugs at the time of sampling were also excluded from the study for both the groups.

### Biochemical analysis

Venous blood samples (6 mL) of all menopausal women with and without CVD were collected after 12-hr overnight fasting under aseptic conditions and were divided into two parts. The blood sample was then centrifuged at 3000 rpm for 15 minutes to obtain a clear serum sample. With the first part (4 mL) biochemical parameters were analyzed. Lipid profile such as serum total cholesterol (TC), triglycerides (TG) and high density lipoprotein-cholesterol (HDL-C) were performed by enzymatic methods. (ERBA kits from Transasia Bio-medicals Ltd., Solan, India) Friedewald formula [10] was used to calculate low density lipoprotein-cholesterol (LDL-C) and very low density lipoprotein-cholesterol (VLDL-C). Serum malondialdehyde (MDA) (Method of Buege and Aust, 1978) [11], LDL carbonyl protein [12] and superoxide dismutase (SOD) (Nandi and Chatterjee, 1998) [13] were estimated as markers of oxidative stress.

### Genotyping

With the second part of blood sample (EDTA, 2 mL) genomic DNA was isolated from intravenous blood by salt precipitation method [14]. Quality of the genomic DNA was checked by agarose gel electrophoresis and quantification of the genomic DNA was done with the help of UV spectrophotometer. The primers were designed by using Software Primer3 (http://simgene.com/primer3). The primers used to amplify were: Forward Primer: 5'-CCTGGTGA-GAAGGGTGAGAA-3'. Reverse primer: 5'-AGAT-GCAGCAAAGCCAAAGT-3'. The PCR cycle involved initial denaturation at 95 °C for 5 minutes followed by 35 cycles of denaturation at 94 °C for 45 seconds, annealing at 50 °C for 45 seconds, primer extension at 72 °C for 45 seconds and final primer extension at 72 °C for 5 minutes. Genotyping of rs1501299 polymorphism in the *AdipoQ* gene was done by polymerase chain reaction (PCR) – based Restriction Fragment Length Polymorphism (RFLP). For validation of this method positive and negative controls were used for every set of reactions. The *AdipoQ* gene polymorphism with respect to Bsm1 recognition sequence is due to guanidine to thymine base pair change which results in loss of Bsm1 restriction site. In case of the presence of Bsm1 cutting site, the amplified DNA fragment was digested (at 60 °C for 90 minutes) into two fragments of 148bp and 93bp which represented homozygous wild (GG) type, uncut single band of 241bp as homozygous mutant (TT) type and three bands of 241bp, 148bp and 93bp as heterozygous (GT) type. The digested amplicons were electrophoresed on 2% agarose gel stained with ethidium bromide to detect the presence/absence of mutation.

### Statistical analysis

**Figure 1:**
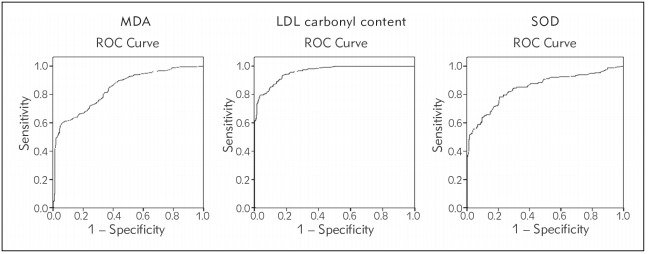
ROC curves of MDA, LDL carbonyl protein and SOD

The statistical analysis was performed using Statistical Package for Social Science program (version 16.0; SPSS Inc., Chicago, IL). Power of the study was calculated by CaTS power calculator [15] and was observed to be more than 80%. For the analysis of MDA, LDL carbonyl protein and SOD levels cut offs were calculated by ROC (Receiving operating characteristic) curves considering sensitivity range as 85-90% and specificity range as 60-90%. The cut off values for MDA, LDL carbonyl protein and SOD were ≤ 1.50 nmol/mL, ≤ 15.04 nmol/mL and > 5.55 units/mL respectively. The area under the ROC curve between 0.8 and 0.9 suggested good precision of diagnostic test (Figure 1). For normal distribution of the data, the normality test was done by Kolmogorov – Smirnov test. The variables not normally distributed (skewed) were log transformed. The continuous data is expressed as mean ± standard deviation (SD). Student's t-test was used to calculate the mean difference between the continuous variables of the two groups. The significant cut-off has been taken as p<0.006 after Bonferroni correction. For data not corrected for Bonferroni, p<0.05 is considered to be significant. Frequencies of genotype and allele in cases and controls were compared using Chi-square (χ^2^) test. Odds ratio (OR) at 95 % confidence interval (CI) were calculated to estimate the risk/protection of *AdipoQ* gene for disease etiology. Logistic regression analysis was performed to determine the risk factors for CVD.

## Results

As shown in Figure 1 the calculated AUC for all the three parameters i.e. MDA, LDL carbonyl protein and SOD, as a single parameter indicated its good clinical precision for oxidative stress. For MDA AUC (95%CI) = 0.842 (0.809 -0.815); SE= 0.017; p<0.001; sensitivity = 85.7% and 1-specificity = 61.2%. Similarly for LDL carbonyl protein AUC (95%CI) = 0.959 (0.945 -0.973); SE= 0.007; p=0.0001; sensitivity = 90.2% and 1-specificity = 84.5%. And for SOD AUC (95%CI) = 0.850 (0.816 -0.883); SE= 0.017; p=0.0001; sensitivity = 85.3% and 1-specificity = 66.9%.

Results presented in Table 1 revealed significant increase (p<0.006) in the levels of Total Cholesterol, Triglycerides, VLDL-C and LDL-C in women with CVD. A significant decrease (p<0.006) in the levels of HDL-C were also observed in women with CVD as compared to women without CVD. Similarly, a marked rise (p<0.006) in the prooxidants level such as MDA and LDL carbonyl protein and a sharp decline (p<0.006) in the antioxidant levels of SOD was observed in CVD group as compared to women without CVD.

**Table 1:** Comparison of clinical characteristics among women with and without CVD *p<0.006 after Bonferroni correction was considered statistically significant. Comparison of lipid profile, prooxidants and antioxidant variables were done using independent t test. Results expressed as Mean ± SD (Standard Deviation). Normality test was done by Kolmogorov – Smirnov. Variables not normally distributed (skewed) were log transformed. VLDL-C, very low density lipoprotein-cholesterol; LDL-C, low density lipoprotein-cholesterol; HDL-C, high density lipoprotein-cholesterol; MDA, malondialdehyde; SOD, superoxide dismutase.

Subjects	Women with CVD (n=265)		Women without CVD (n=258)		p value
Variables	Range	Mean ± SD	Range	Mean ± SD	
Total Cholesterol (mmol/L)	3.80 – 9.11	5.80 ± 1.14	3.67 – 7.12	5.29 ± 0.83	0.0001*
Triglycerides (mmol/L)	1.26 – 4.49	2.10 ± 0.29	1.0 – 3.18	1.79 ± 0.20	0.0001*
VLDL-C (mmol/L)	0.24 – 0.89	0.40 ± 0.06	0.20 – 0.63	0.34 ± 0.04	0.0001*
LDL-C (mmol/L)	2.09 – 7.04	3.91± 1.03	2.04 – 5.12	3.24 ± 0.69	0.0001*
HDL-C (mmol/L)	0.46 – 1.29	0.92 ± 0.18	0.72 – 1.70	1.20 ± 0.16	0.0001*
MDA (nmol/mL)	1.01 – 3.83	2.18 ± 0.60	1.0 – 3.3	1.41 ± 0.2	0.0001*
LDL carbonyl content (nmol/mL)	10.68 – 39.18	24.87 ± 7.00	6.0 – 26.3	10.71 ± 1.75	0.0001*
SOD (units/mL)	1.0 – 12.9	2.75 ± 1.2	2.0 – 15	7.00 ± 2.79	0.0001*

As shown in Table 2 distribution of homozygous TT genotype was quite higher among cases (20.4%) than among controls (2.7%). The comparison of frequency distribution of alleles and genotypes of *AdipoQ* (rs1501299) polymorphism in women of Punjab showed that the frequency of minor T allele was also found to be higher in cases (35.5%) than in controls (29.5%). Statistical significant difference (p<0.05) was observed in the distribution of allele (p=0.04) as well as genotypes (p=0.001) with T allele conferring risk towards CVD susceptibility.

**Table 2:** Genotype and allele frequency distribution of *AdipoQ* (rs1501299) polymorphism in women with and without CVD *p<0.05 is statistically significant

Genotype/ allele frequency	Total women		Normolipidemic women		Hyperlipidemic women	
	With CVD n = 265	Without CVD n = 258	With CVD n = 99	Without CVD n = 142	With CVD n = 166	Without CVD n = 116
GG	49.4	43.8	43.5	45.8	53.0	41.4
GT	30.2	53.5	32.3	51.4	29.0	56.0
TT	20.4	2.7	24.2	2.8	18.0	2.6
	p = 0.001*		p = 0.001*		p = 0.001*	
G	64.5	70.5	59.6	71.5	67.5	69.4
T	35.5	29.5	40.4	28.5	32.5	30.6
	p = 0.041* OR = 1.31 (1.01–1.70)		p = 0.008* OR = 1.69 (1.15–2.49)		p = 0.695 OR = 1.09 (0.76–1.57)	
GT+TT Dominant model (GT+TT vs GG)	50.6 56.2 p = 0.228 OR = 0.79 (0.56–1.12)		56.5 54.2 p = 0.819 OR = 1.09 (0.65–1.84)		47.0 58.6 p = 0.071 OR = 0.62 (0.38–1.01)	
GT+GG Recessive model (TT vs GT+GG)	79.6 97.3 p = 0.001* OR = 9.17 (4.08–20.59)		75.8 97.2 p = 0.001* OR = 11.04 (3.69–33.01)		82.0 97.4 p = 0.001* OR = 8.30 (2.47–27.9)	
GG+TT Co-dominant model (GT vs GG+TT)	69.8 46.5 p = 0.001* OR = 0.37 (0.26–0.53)		67.7 48.6 p = 0.005* OR = 0.45 (0.26–0.77)		71.0 44.0 p = 0.001* OR = 0.31 (0.19–0.52)*	

Under recessive model (TT vs GT+GG) analysis TT homozygotes provided significant (p=0.001) risk ∼9 folds towards CVD predisposition. Dominant model (GT+TT vs GG) revealed no significant difference (p=0.228) in the distribution while, co-dominant model (GT vs GG+TT) provided protection (p=0.001) for CVD when women with and without CVD were compared.

Analysis of *AdipoQ* (rs1501299) polymorphism for distribution of genotype and allele frequencies was further categorized into normolipidemics and hyperlipidemics according to the lipidemic status. The criterion for labeling the subjects under the category of normolipidemics and hyperlipidemics was taken according to the levels recommended in National Cholesterol Education Program (NCEP) ATP III guidelines [16].

The frequency of risk T allele in normolipidemic cases was observed to be significantly (p=0.008) more (40.4%) than in normolipidemic controls (28.5%). Recessive model (TT vs GT+GG) analysis showed ∼ 11 fold high risk (p=0.001) towards CVD predisposition where as codominant model (GT vs GG + TT) revealed protection (p=0.005) for CVD. On the contrary dominant model (GT+TT vs GG) revealed insignificant difference (p=0.819) when cases were compared with controls.

Significant difference (p<0.05) was also observed in the frequency distribution of genotype (p=0.001) among hyperlipidemic cases and controls. Recessive model (TT vs GT+GG) analysis revealed ∼8 fold increased risk (p=0.001) towards CVD susceptibility and codominant model (GT vs GG+TT) revealed protection towards CVD (p=0.001). However, dominant model (GT + TT vs GG) revealed insignificant difference (p=0.071) when hyperlipidemic cases were compared with that of controls.

The subjects were further categorized according to the cut offs for the variables of oxidative stress that is MDA, LDL carbonyl protein and SOD. The comparison of frequency distribution of allele and genotypes among the total women with and without CVD for MDA>1.50 in Table 3 revealed that minor T allele frequency was significantly (p=0.030) more (36.6%) in cases than in controls (27.5 %). Under recessive model (TT vs GT+GG) T allele carriers conferred 9∼10 folds high risk (p=0.001) towards CVD susceptibility and codominant model provided protection (p=0.001) for CVD in women with MDA >1.50. On the contrary, dominant model (GT+TT vs GG) revealed insignificant difference (p=0.859) on comparison between cases and controls.

**Table 3:** Stratified analysis of *AdipoQ* (rs1501299) genotypes with oxidative stress in women with and without CVD *p<0.05 was considered statistically significant. DM: dominant model; RM: recessive model; CDM: co-dominant model

Subjects		Genotypes/Allele/Models	With CVD n (%)	Without CVD n (%)	p value	OR (95% CI)
Variables						
MDA (nmol/mL)	>1.50	GG	113 (49.8)	48 (48)	0.001*	
		GT	62 (27.3)	49 (49)		
		TT	52 (22.9)	03 (3)		
		G	288 (63.4)	145 (72.5)	0.030*	1.52 (1.05–2.18)
		T	166 (36.6)	55 (27.5)		
		DM			0.859	0.93 (0.58–1.49)
		RM			0.001*	9.60 (2.92–31.58)
		CDM			0.001*	0.39 (0.23–0.63)
LDL carbonyl protein (nmol/mL)	>15.04	GG	113 (47.3)	16 (40)	0.001*	
		GT	73 (30.5)	23 (57.5)		
		TT	53 (22.2)	01 (2.5)		
		G	299 (62.6)	55 (68.8)	0.347	1.31 (0.79–2.18)
		T	179 (37.4)	25 (31.2)		
		DM			0.494	0.74 (0.37–1.47)
		RM			0.007*	11.11 (1.49–82.83)
		CDM			0.001*	0.32 (0.16–0.64)
SOD (units/mL)	≤ 5.55	GG	112 (49.6)	39 (45.9)	0.001*	
		GT	71 (31.4)	44 (51.8)		
		TT	43 (19)	02 (2.3)		
		G	295 (65.3)	122 (71.8)	0.149	1.35 (0.91–1.99)
		T	157 (34.7)	48 (28.2)		
		DM			0.652	0.86 (0.52–1.42)
		RM			0.001*	9.75 (2.30–41.22)
		CDM			0.001*	0.42 (0.25–0.71)
	> 5.55	GG	19 (48.8)	74 (42.8)	0.001*	
		GT	09 (23.0)	94 (54.3)		
		TT	11 (28.2)	05 (2.9)		
		G	47 (60.3)	242 (70)	0.127	1.53 (0.92–2.55)
		T	31 (39.7)	104 (30)		
		DM			0.619	0.78 (0.39–1.57)
		RM			0.001*	13.20 (4.26–40.88)
		CDM			0.001*	0.25 (0.11–0.56)

For LDL carbonyl protein>15.04 under recessive model (TT vs GT+GG) analysis TT genotype conferred ∼11 fold high risk (p=0.007) towards CVD susceptibility and GT genotype provided protection (p=0.001) for CVD when cases were compared with controls. On the contrary, dominant model (GT+TT vs GG) analysis revealed insignificant difference (p=0.494) on comparison between cases and controls.

Comparison between the subjects with and without CVD revealed that T allele carriers of recessive model (TT vs GT+GG) conferred 9∼10 folds high risk towards CVD susceptibility (p=0.001). Codominant model (GT vs GG+TT) provided protection for CVD (p=0.001) and dominant model (GT+TT vs GG) showed insignificant difference (p=0.652) among the subjects with SOD≤5.55.

For SOD>5.55, under recessive model (TT vs GT+GG) analysis T allele carriers conferred ∼13 fold increased risk towards CVD predisposition (p=0.001) and codominant model (GT vs GG+TT) analysis provided protection for CVD (p=0.001). However, dominant model (GT+TT vs GG) analysis revealed no statistical significant difference (p=0.619) among the women with and without CVD for SOD>5.55.

When these subjects were further categorized into normolipidemics and hyperlipidemics (Table 4), stratified analysis of *AdipoQ* (rs1501299) revealed significant difference for both normolipidemic (p=0.003) and hyperlipidemic (p=0.002) TT genotypes as compared to GG and GT genotypes with CVD for MDA >1.50 when cases were compared with control.

**Table 4:** Stratified analysis of *AdipoQ* (rs1501299) genotypes with oxidative stress in normolipidemic and hyperlipidemic women with and without CVD. *p<0.05 was considered statistically significant. DM: dominant model; RM: recessive model; CDM: co-dominant model

Subjects		Genotypes/Allele/Models	Normolipidemic Women		Hyperlipidemic Women	
Variables			With CVD n (%)	Without CVD n (%)	With CVD n (%)	Without CVD n (%)
MDA (nmol/mL)	>1.50	GG	38 (43.7)	25 (48)	75 (53.6)	23 (48)
		GT	25 (28.7)	25 (48)	37 (26.4)	24 (50)
		TT	24 (27.6)	02 (4)	28 (20.0)	01 (2)
			p = 0.003*		p = 0.002*	
		G	101 (58)	75 (72.1)	187 (66.8)	70 (72.9)
		T	73 (42)	29 (27.9)	93 (33.2)	26 (27.1)
			p = 0.026* OR = 1.87 (1.10–3.15)		p = 0.323 OR = 1.33 (0.80–2.24)	
LDL carbonyl protein (nmol/mL)	>15.04	GG	41 (42.7)	07 (41.2)	72 (50.3)	09 (39.1)
		GT	31 (32.3)	10 (58.8)	42 (29.4)	13 (56.6)
		TT	24 (25.0)	00	29 (20.3)	01 (4.3)
			p = 0.071		p = 0.052	
		G	113 (58.9)	24 (70.6)	186 (65.0)	31(67.4)
		T	79 (41.1)	10 (29.4)	100 (35.0)	15 (32.6)
			p = 0.271 OR = 1.67 (0.76–3.70)		p = 0.885 OR = 1.11 (0.57–2.15)	
SOD (units/mL)	≤5.55	GG	36 (42.4)	22 (47.8)	76 (53.9)	17 (43.6)
		GT	29 (34.1)	23 (50)	42 (29.8)	21 (53.8)
		TT	20 (23.5)	01 (2.2)	23 (16.3)	01 (2.6)
			p = 0.012*		p = 0.016*	
		G	101 (59.4)	67 (72.8)	194 (68.8)	55 (70.5)
		T	69 (40.6)	25 (27.2)	88 (31.2)	23 (29.5)
			p = 0.042* OR = 1.35 (0.91–1.99)		p = 0.878 OR = 1.08 (0.62–1.87)	
	>5.55	GG	07 (50)	43 (44.8)	12 (48)	31 (40.3)
		GT	03 (21.4)	50 (52.1)	06 (24)	44 (57.1)
		TT	04 (28.6)	03 (3.1)	07 (28)	02 (2.6)
			p = 0.005*		p = 0.001*	
		G	17 (60.7)	136 (70.8)	30 (60)	106 (68.8)
		T	11 (39.3)	56 (29.2)	20 (40)	48 (31.2)
			p = 0.385 OR = 1.57 (0.69–3.56)		p = 0.327 OR = 1.47 (0.76–2.85)	

Insignificant difference was observed among the genotypes with LDL carbonyl protein >15.04 when cases were compared with controls for both normolipidemics and hyperlipidemics.

Significant difference was observed among normolipidemic (p=0.012) as well as hyperlipidemic (p=0.016) genotypes for SOD≤5.55. Similarly, significant difference was also observed among normolipidemic (p=0.005) as well as hyperlipidemic (p=0.001) genotypes for SOD>5.55 when cases were compared with controls.

Logistic regression analysis was further performed to understand the potential association of various risk factors with CVD as dependant variable. As shown in Table 5 after applying logistic regression it has been observed that MDA, LDL carbonyl protein and SOD were strongly correlated with CVD after adjusting for menopausal age, BMI, WC and WHR. TT homozygotes for *AdipoQ* (rs1501299) gene polymorphism also conferred ∼5 fold increase of cardiovascular risk. However, heterozygosity at *AdipoQ* (1501299) gene polymorphism provided protection for CVD. Whereas, rs 1501299 was not associated with CVD in homozygote GG model.

**Table 5:** Logistic regression analysis of women with CVD and associated risk factors *p<0.05 for MDA, LDL carbonyl protein and SOD after adjusting for confounding factors such as menopausal age, BMI, WC and WHR. Normality test was done by Kolmogorov – Smirnov. Variables not normally distributed (skewed) were log transformed.

Variables	p value	Odds Ratio (OR)	95% CI
MDA	0.001*	4.10	2.12 – 7.93
LDL carbonyl protein	0.001*	21.82	11.37 – 41.85
SOD	0.001*	11.77	7.63 – 18.17
TT genotype (rs1501299)	0.013*	5.22	1.41 – 19.32
GT genotype (rs1501299)	0.001*	0.29	0.14 – 0.58
GG genotype (rs1501299)	0.056	1.80	0.98 – 3.30

## Discussion

Lipid peroxidation and LDL oxidation induced by the reactive oxygen species are the early event in atherosclerotic lesion formation [17]. Malondialdehyde (MDA) which is one of the products of lipid peroxidation has been the most extensively studied marker. Its increased level marks the index of assessing oxidative stress [18]. Accordingly, significant difference in the mean levels of MDA as a marker of oxidative modification in lipid was observed, when cases were compared to that of controls in the present study. Similarly, significant increased levels of MDA were also observed by Sowmya et al. [19]. Another study by Bhargava et al. [20] reported increased levels of MDA in patients with CAD and CVD with hyperhomocystenemia. Similarly, in our previous report, we had observed increased levels of MDA in menopausal women suffering from CVD [21]. On the contrary, Lopes et al. [22] observed no difference in the concentration of MDA between atherosclerotic subjects and controls.

For another studied prooxidant LDL carbonyl protein, significant difference in the mean levels was again observed in subjects with CVD as compared to subjects without CVD. Similarly, increased levels of carbonyl protein were also observed in the patients of CVD in our earlier reported study [21] and in a study conducted by Lopes et al. [22]. In another study by Jawalekar et al. [23] increase in protein carbonyl in red cell extract in the patients with IHD, HTN and cerebrovascular disease as compared to the healthy controls were reported. This suggests that elevated levels of protein carbonyls might be associated with clinical complications.

Extracellular SOD (ec-SOD) is a secretary glycoprotein and a powerful antioxidant whose levels are found high in blood vessels so as to suppress oxidative stress under normal conditions. The present study findings substantiate this theory since it was found that increased lipid peroxidation was associated with decreased activity of the endogenous antioxidant enzyme SOD. Present study revealed decreased mean levels of SOD in the subjects with CVD as compared to subjects without CVD. This decrease in SOD activity may be due to the effect of increased oxygenderived free radicals on SOD. Because, superoxide anion which is the main oxygen species reacts with nitric oxide radical forming peroxynitrite – a free radical, causing oxidative stress and cellular damage. Gumanova et al. [24] in their study found that higher levels of NO are significantly associated with coronary lesions though, some studies reported increased activity of SOD in ischemic heart disease patients [25]. On the other hand, other studies similar to our study revealed decreased activity of SOD [19] [21] [26].

The comparison of allele frequency for *AdipoQ* (1501299) polymorphism with other world populations revealed that in the present Punjabi population, minor allele frequency (MAF) for *AdipoQ* gene reported was quite similar (29.5%) to that reported in Japanese population (29.7%) [27]; Greek women (28%) [28]; and in Chinese adolescents (30.6%) [29]. Under recessive model analysis TT genotype of *AdipoQ* (rs1501299) polymorphism provided a significant risk ∼9 folds towards CVD predisposition.

In the present studied population with CVD, the frequency of T allele carrier was more as compare to subjects without CVD. Similar, to the present study Fillipi et al. [30] observed increased risk of CAD with T allele in Italian population. Study by Ghattas et al. [31], also indicated that TT genotype of *AdipoQ* (rs1501299) polymorphism was associated with increased risk of CAD in Egyptian population. In another study by Tong et al. [32] on Chinese population the *AdipoQ* (rs1501299) variant was also positively related with an increased risk of CAD. Mohammadzadeh et al. [33] revealed on their studied population of Iranians that both GT and TT genotype along with T allele of SNP rs1501299 were related to increased risk of CAD. Furthermore, they also observed that frequency of T allele in female CAD patients was significantly more than that of control subjects. On the contrary, Qi et al. [34] reported that rs1501299 was significantly associated with a ∼45% decreased CVD risk in females from Nurses' Health Study. Decreased risk in homozygous carriers of T allele compared with carriers of G allele was also observed in Caucasian population by Bacci et al. [35].

Associations of various candidate genes with predisposition to CVD have been extensively reported and polymorphism at multiple genes has been coupled with differential effects of lipid metabolism with this disease [21]. Adiponectin attenuates expression of class A scavenger receptor in human macrophages and inhibits transformation of macrophages to foam cells [36]. The modified LDL particles do not bind readily to the endogenous LDL receptor and are therefore not cleared from the circulation by this mechanism. They enter the arterial intima more easily and are more easily oxidized, probably because they contain less antioxidant protection. Oxidized LDL consists of numerous modified lipid and protein molecules. They are thus, taken up by the macrophage scavenger receptors accelerating foam-cell formation [37]. In the present study, when the subjects were divided according to the lipidemic status into normolipidemics and hyperlipidemics, prevalence of T allele was more in cases as compared to controls. Interestingly, carriers of T normolipidemic homozygotes with CVD under recessive model conferred more risk (∼11 folds) towards CVD susceptibility as compared to hyperlipidemic (∼8 folds) subjects with CVD on comparison with that of controls. This indicates that event of cardiovascular disease starts earlier even when lipids are normal, thus signifying the involvement of oxidative stress as an early event in the atherosclerotic process, as also proved by other studies [18] [38] [39]. Thus, in the present studied population we observed that not only hyperlipidemic TT homozygotes but also normolipidemic TT homozygotes were related to an increased risk of CVD.

Oxidative stress is the main regulator of various signal transduction systems involved in atherosclerotic vascular inflammation and throughout the whole formation process of fatty streaks and lesions progressing to final plaque rupture [40]. At cellular and molecular level adipo has anti-inflammatory antioxidant and anti-apoptotic roles, thereby mitigating key mechanisms underlying CVD pathogenesis. Increased oxidative stress and decreased antioxidant activity or both are major mechanism(s) involved in the pathogenesis of cardiovascular disease. Oxygenfree radicals are generated particularly in the early stages of MI and the decrease in SOD activity may be due to effect of increased oxygen – derived free radicals in SOD. Thus, these results suggest that oxidative stress may affect the initiation and progression of atherogenesis in CVD patients.

### Limitation

The lacuna of the study is that we could not measure the levels of adiponectin in blood since its analysis was beyond the scope of the present study. Measurement of adiponectin levels in blood and its association with markers of oxidative stress can throw more light on the role of *AdipoQ* (rs1501299) gene in the occurrence of cardiovascular disease.

## Conclusion

On the basis of observed results the present study reveals that rs1501299 variation in *AdipoQ* gene is among the genetic factor predisposing population of Punjab to CVD. The women of Punjab (India) who are homozygous for T allele of *AdipoQ* gene have been observed to have more oxidative stress and hence, they are at higher risk of CVD than carriers of G allele. Therefore, screening of this studied polymorphism along with other associated tagSNPs can help in the prognosis/diagnosis of CVD pathophysiology. However, the functional implication is yet to be evaluated. Moreover, additional studies and larger study groups are needed to confirm and strengthen the current results that may correlate with this SNP and have a clearer function. Cardiovascular health awareness is extremely important for primary prevention of the disease among menopausal women. Scrutinizing oxidative stress parameters and genetic markers along with other routine biochemistry investigations may play a fundamental role in diagnostic process of CVD at the earlier stage.

## Acknowledgments

The authors are thankful to all the study participants who consented to be part of this study and are also grateful to Professor Parneet Dhillon, Department of English of Khalsa College Amritsar for vetting the paper with respect to grammatical mistakes.

## Conflict of interest statement

All the authors declare that they have no conflict of interest in this work.
